# Forest Fruit Production Is Higher on Sumatra Than on Borneo

**DOI:** 10.1371/journal.pone.0021278

**Published:** 2011-06-28

**Authors:** Serge A. Wich, Erin R. Vogel, Michael D. Larsen, Gabriella Fredriksson, Mark Leighton, Carey P. Yeager, Francis Q. Brearley, Carel P. van Schaik, Andrew J. Marshall

**Affiliations:** 1 Anthropological Institute, University of Zurich, Zurich, Switzerland; 2 Sumatran Orangutan Conservation Programme (PanEco-YEL), Medan, Sumatra, Indonesia; 3 The Center for the Advances Study of Hominid Paleobiology, The George Washington University, Washington D. C. United States of America; 4 Department of Statistics, The George Washington University, Washington, United States of America; 5 Institute for Biodiversity and Ecosystem Dynamics/Zoological Museum, University of Amsterdam, Amsterdam, The Netherlands; 6 Great Ape World Heritage Species Project, Babson Park, Massachusetts, United States of America; 7 Zoology Department, University of Florida, Gainesville, Florida, United States of America; 8 School of Science and the Environment, Manchester Metropolitan University, Manchester, United Kingdom; 9 Graduate Group in Ecology, and Animal Behaviour Graduate Group, Department of Anthropology, University of California Davis, Davis, California, United States of America; Centre National de la Recherche Scientifique, France

## Abstract

**Background:**

Various studies have shown that the population densities of a number of forest vertebrates, such as orangutans, are higher on Sumatra than Borneo, and that several species exhibit smaller body sizes on Borneo than Sumatra and mainland Southeast Asia. It has been suggested that differences in forest fruit productivity between the islands can explain these patterns. Here we present a large-scale comparison of forest fruit production between the islands to test this hypothesis.

**Methodology/Principal Findings:**

Data on fruit production were collated from Sumatran and Bornean sites. At six sites we assessed fruit production in three forest types: riverine, peat swamp and dryland forests. We compared fruit production using time-series models during different periods of overall fruit production and in different tree size classes. We examined overall island differences and differences specifically for fruiting period and tree size class. The results of these analyses indicate that overall the Sumatran forests are more productive than those on Borneo. This difference remains when each of the three forest types (dryland, riverine, and peat) are examined separately. The difference also holds over most tree sizes and fruiting periods.

**Conclusions/Significance:**

Our results provide strong support for the hypothesis that forest fruit productivity is higher on Sumatra than Borneo. This difference is most likely the result of the overall younger and more volcanic soils on Sumatra than Borneo. These results contribute to our understanding of the determinants of faunal density and the evolution of body size on both islands.

## Introduction

Patterns of fruit production vary substantially among tropical forests. Studies of tropical forest phenology have demonstrated differences in fruit production between continents [1], [2], between sites [3]–[6], and between forest types within sites [7]–[13]. This variation is thought to be determined by a range of factors, such as soil nutrients [9], [10], [14]–[18], rainfall [4], [19], [20], latitude [21], altitude [7], [8], [21], levels of carbon dioxide [22], [23], and solar irradiation [1], [24], [25]. Characterizing differences in fruit production at different spatial scales may shed light on a range of questions in forest and vertebrate ecology, vertebrate evolution, biogeography, and conservation biology. For example, fruit productivity is typically thought to set carrying capacity for rain forest vertebrates, which in turn may have effects on plant life history, seed dispersal, and vertebrate population dynamics [16], [26]–[31]. In addition, resource availability differences on islands might affect the evolution of mammal body size [32].

Many studies of tropical forest phenology have been conducted in Malesia (e.g. [5], [33]–[35]), in part due to the unusual patterns of inter-specific gregarious fruiting characteristic of the region (i.e., mast fruiting: [26], [36]–[38]). Despite the existence of a number of long-term data sets, few attempts have been made to compare general patterns of fruit production in different parts of Malesia (but see [5], [33], [39] for comparisons of mast fruiting across sites). It has been suggested that Sumatran rain forests are generally more productive than their Bornean counterparts [39]–[41]. This hypothesis is based on the assumption that ongoing tectonic activity, including uplift, and recent volcanism has created more fertile soils on much of Sumatra than are found on most of Borneo [41], [42]. This region is well-suited for a comparative study because the forests on Sumatra and Borneo have recurrently been connected during the various glacial periods, which has led to mixing of both plant and animal species (e.g. [43]). Hence, systematic differences between Borneo and Sumatra in for instance plant productivity and subsequent differences mammal body size are more likely to be the result of relatively short-term environmental differences such as differences in soil nutrition than long-term differences (allopatric divergence). In addition, the observation that primate biomass is higher on Sumatra than on Borneo suggests that Sumatran forests may be more productive [44]. There are also several differences in the behavioural ecology and life history of orangutans (*Pongo* spp.) that are thought to have resulted from higher fruit production on Sumatra than Borneo [40], [45]–[47]. In addition, the lower productivity in Borneo has been hypothesised to influence the reduction in body size of several mammal species. Body sizes of the Malayan Sun Bear (*Helarctos malayanus*: [48]), the greater chevrotain (*Tragulus napu*: [49]), sambar (*Cervus unicolor*: [50]) and many others [51] are all smaller on Borneo compared to Sumatra and mainland Asia. Moreover, Meiri *et al.* [52] found that among an entire ecological guild, the carnivores, Bornean forms were smaller than their mainland conspecifics. Such smaller size evolution could have been the result of lower resource availability [32] and it is thus important to determine whether resource availability indeed is lower on Borneo.

In this paper we compare fruit productivity between forests on the islands of Sumatra and Borneo using a standard measure, the percentage of trees per month that carry fruit. This measure has been used in several studies of forest and vertebrate ecology (e.g. [5], [35], [53]). We hypothesise that on average Sumatran forests have a higher fruit production than forests on Borneo, but that this will only occur in those Sumatran forests that potentially have nutrient influences from fertile soils. Thus Sumatran forests that are fed nutrients from rivers or nutrient run-off from large mountain massifs should show higher fruit production, but those Sumatran forests that do not obtain such nutrient influences are predicted to be similar in productivity to Bornean forests.

## Results

### Overall Island Differences

**Figure 1:**
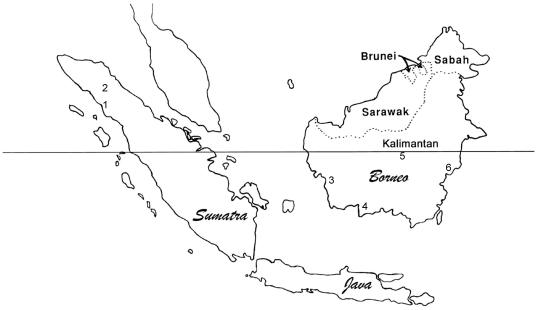
Site locations. Locations of study sites: 1 = Suaq; 2 = Ketambe; 3 = Gunung Palung; 4 = Tanjung Puting; 5 = Barito Ulu; 6 = Sungai Wain.

The time-series model of fruit productivity accounting for variation in DBH and fruit level category resulted in a significant difference in fruit production between Borneo and Sumatra (overview of sites: Table 1, Figure 1), with Sumatra being characterized as having significantly higher levels of fruit production (Table 2). To further illustrate these differences, we break each analysis down by forest type (Table 3).

**Table 1:** Habitat characteristics and survey effort at each site.

Sites	Island	Altitude (m)	Annual rainfall (mm)	Total trees	# trees 15–29.9 cm dbh	# trees 30–44.9 cm dbh	# trees 45–59.9 cm dbh	# trees 60–74.9 cm dbh	# trees 75–89.9 cm dbh	# trees >90 cm dbh	Sample area (ha)	Data collection Period	# months
*Peat forest*													
Suaq	S	5	3362	424	278	86	35	14	3	8		Feb 94–Aug 99	66
GP	B	5–10	4300	779	487	181	74	20	11	6	1.5	Jan 86–Sep 91	79
TP	B	5–10	3026	891	666	181	32	8	4	0	2.0	Apr 93–Jun 96	40
*Riverine forest*													
Suaq	S	5	3362	183	100	47	16	9	4	7		Feb 94–Aug 99	66
GP	B	5–10	4300	890	608	168	69	25	15	5	2.0	Jan 86–Sep 91	79
*Dryland forest*													
Ketambe	S	350–500	3288	600	387	110	56	17	14	16	1.1*	Sep 88–May 01	153
Suaq	S	5–150	3362	309	194	55	29	10	6	15		Feb 94–Aug 99	67
Sungai Wain	B	30–150	2968	315	233	46	17	13	0	6	1.0	Jan 98–Jul 02	55
Barito Ulu	B	100–300	3750	134	107	18	7	1	1	0	-	Nov 90–Jun 00	124
GP AB	B	5–50	4300	718	413	168	72	30	16	19	2.0	Jan 86–Sep 91	79
GP LS	B	20–200	4300	1139	614	280	98	67	30	50	3.0	Jan 86–Sep 91	79
GP	B	200–400	4300	934	472	273	95	57	20	17	1.8	Jan 86–Sep 91	79

Note: S = Sumatra, B = Borneo, GP = Gunung Palung, AB = alluvial bench, LS = lowland sandstone, LG = lowland granite forest, TP = Tanjung Puting. For some sites there is no sample area as the area in which the trees were located was not measured. Rainfall data from the references in the method section for each site except for Tanjung Puting [75].

*in Ketambe total plot area was extended to 1.6 ha in 1997.

**Table 2:** Differences in time series estimated fruit production between Borneo and Sumatra.

	Mean difference in % fruiting (Sumatra-Borneo)	SE	t-statistic	P-value (two-sided)
All sites combined	13.06	0.22	59.54	<0.0001
Dryland forests	10.90	0.26	41.18	<0.0001
Peat swamp forests	23.84	0.53	45.35	<0.0001
Riverine forests	6.08	0.49	12.51	<0.0001

Note: All sites are combined and split by habitat type. Models include estimates for DBH and fruit level category. SE is standard error.

**Table 3:** Pairwise comparisons of all sites within Borneo and Sumatra by habitat.

Bornean site	Sumatran Site	Habitat type	Estimated Mean % (Borneo)	Estimated Mean % (Sumatra)	Mean difference in % fruiting (Sumatra-Borneo)	SE	t-value	DF	p-value	Adjusted p-value
BU	KET	Dry	5.41	25.11	19.7	0.5	39.65	835	<0.0001	<0.0001
GP AB	KET	Dry	6.59	25.11	18.53	0.4	46.75	1147	<0.0001	<0.0001
GP LG	KET	dry	3.97	25.11	21.14	0.62	34.3	951	<0.0001	<0.0001
GP LS	KET	dry	6.49	25.11	18.62	0.4	46.19	1195	<0.0001	<0.0001
SW	KET	dry	3.03	25.11	22.08	0.4	54.64	1096	<0.0001	<0.0001
BU	SB	dry	5.41	6.89	1.48	0.43	3.41	497	0.0007	0.009
GPAB	SB	dry	6.59	6.89	0.3	0.31	0.97	495	0.3344	1
GPLG	SB	dry	3.97	6.89	2.92	0.57	5.16	687	<0.0001	<0.0001
GPLS	SB	dry	6.49	6.89	0.4	0.32	1.23	536	0.2177	1
SW	SB	dry	3.03	6.89	3.86	0.32	11.97	489	<0.0001	<0.0001
GP PS	SB	peat	6.81	30.57	23.76	0.59	40.48	443	<0.0001	<0.0001
TNPUT	SB	peat	6.65	30.57	23.92	0.52	45.96	296	<0.0001	<0.0001
GPFS	SB	river	6.08	12.17	6.08	0.49	12.51	373	<0.0001	<0.0001

Note: Time series estimated fruit production means (% fruiting). Models include DBH and fruit level category as variables in the model. (For Gunung Palung (GP), AB = alluvial bench, LS = lowland sandstone, LG = lowland granite, PS = peat swamp, FS = freshwater swamp). SE is standard error. DF is degrees of freedom. P-values are two-sided. The adjusted p-value is the maximum of 1 and the p-value times 13.

### Island Variation among Habitat Types

**Figure 2:**
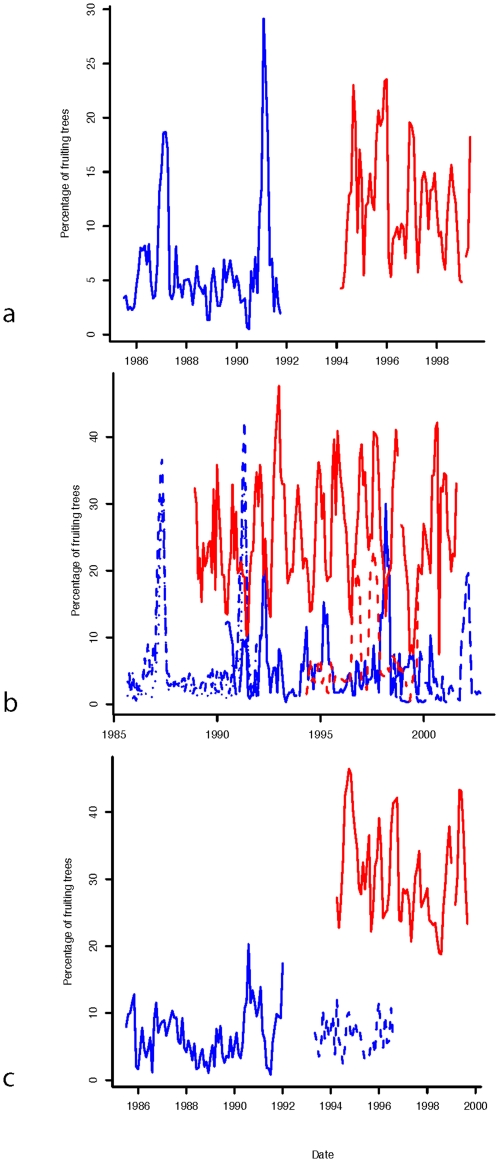
Graph of forest fruit availability. Time series graphs of each forest type (Fig 2a, b, c) (Riverine (a), Dryland forest (b), Peat (c). Blue = Borneo, Red = Sumatra.

**Figure 3:**
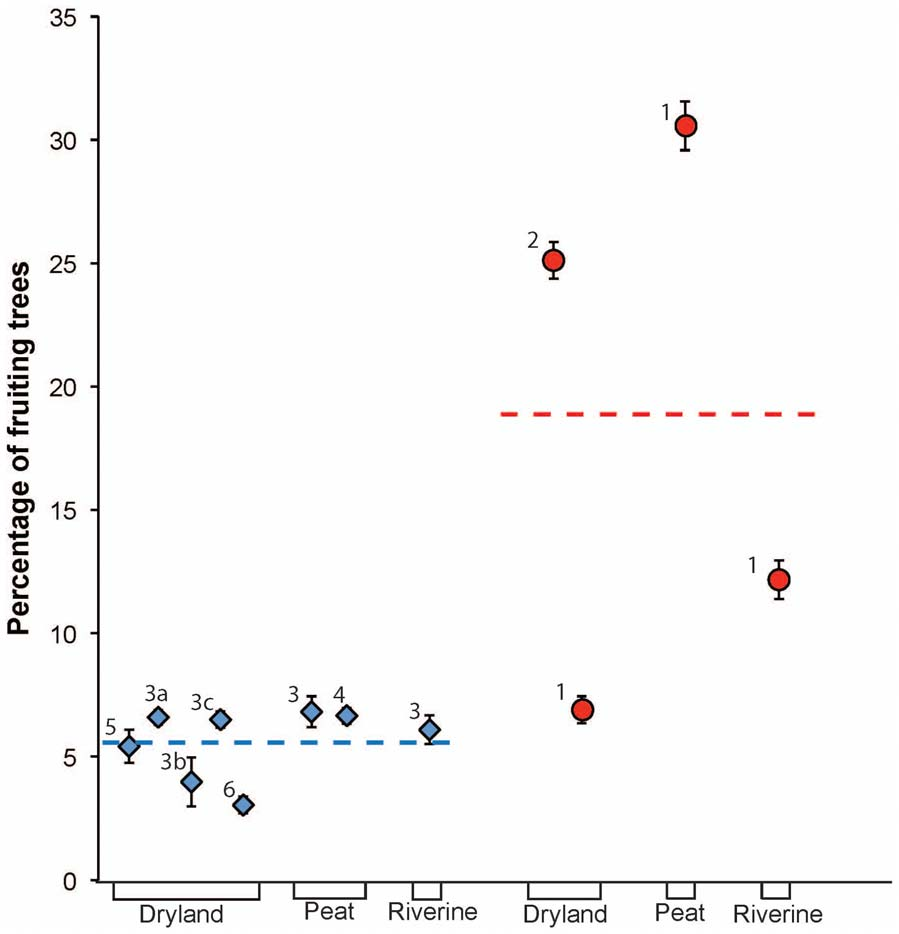
Mean fruit availability per site. Graph of time-series corrected model mean estimates of the percentage of fruiting trees and 95% confidence intervals for all sites in Borneo and Sumatra separated by habitat type. Field sites in Borneo are represented by blue triangles and those in Sumatra are represented by red circles. The numbers in the figure correspond to the following field areas or sites: 1 = Suaq Balimbing, 2 = Ketambe, 3 = Gunung Palung, 3a = Gunung Palung AB, 3b = Gunung Palung LG, 3c = Gunung Palung LS, 4 = Tanjung Puting, 5 = Barito Ulu, 6 = Sungai Wain.

Riverine and dryland forest habitats showed the greatest fluctuations in fruit production, with high peaks characteristic of mast fruiting (Figures 2a, b), while fruit production in peat swamp forests showed less pronounced fluctuation (Figure 2c). Overall, fruit production was consistently higher in Sumatra compared to Borneo across all three habitat types (Figures 2, 3; Riverine: Table 3 and Methods S1, Table S1, S2; Peat Swamp Forest: Table 3 and Tables S3, S4, S5, S6; Dryland Forest: Table 3, Tables S7, S8). This pattern held across most DBH classes and fruiting periods, with the few exceptions occurring primarily for smaller DBH classes during low fruit periods (Tables S2, S4, S5, S6, S8). The only cases where a Bornean forest had higher overall fruit production was for the dryland forest habitats of Gunung Palung AB and Gunung Palung LS compared to Suaq Balimbing in Sumatra (Table 3).

## Discussion

The results in this paper clearly show differences in fruit production among sites. It is, however, important to determine whether these differences vary systematically between the two islands and therefore support our hypothesis or merely reflect site differences that are unrelated to island differences. Overall, fruit production in the three forest types assessed is higher on Sumatra than on Borneo (Table 2). For riverine and peat swamp forests, Suaq (Sumatra) shows a significantly higher fruit production than the Bornean sites. For dryland forests it is clear that Ketambe and Suaq (Sumatra) have a significantly higher fruit production than almost all forests on Borneo. Only for two of the pairwise comparisons did a Sumatran site (Suaq) not show a significantly higher fruit production than a Bornean site (Gunung Palung). The Suaq dryland forest is somewhat unique in that it is a completely isolated low lying hill that does not obtain any nutrient influence from the Leuser mountain massif as the forest at Ketambe does and as do most forests in the area. This nutrient influence for forests such as Ketambe that are located at the base or on slopes of large mountain massifs might explain why some Sumatran sites appear to have such a high fruit production and, at even high altitude, they still show high orangutan densities [31]. Thus although the general trend is that there is an overall higher productivity in Sumatra, it is important to realise that not all inter-site comparisons follow this general trend. More long-term phenological data, especially from Sumatran sites, are needed to examine the general applicability of the trends indicated here. Ideally new studies on both islands should use similar phenological data collection methods that record both fruiting frequency and fruit crop sizes. Such refinements are needed to fully appreciate potential differences between sites and islands.

Because the number of sites is limited it is relevant to assess whether the pattern found here with the two Sumatran sites having a higher fruit production than the four Bornean sites. Under the assumption that all sites are equal, the chance that the two Sumatran sites would be higher than the four Bornean sites is 0.067. This is a very conservative probability because it is purely based on ranking and does not incorporate the real fruit production percentages, which would lead to lower p-values. If one would use the 12 locations instead of the six sites the ranking found has a probability of 0.008. Thus the chance that the pattern found is due to chance is low, which gives confidence into the generality of these results.

It is also important to determine whether there are overall factors that potentially differ between the islands and therefore can explain the variation. Several studies have shown that rainfall is correlated to plant productivity (e.g. [4]). Mean annual rainfall at the Sumatran sites seems similar to that for the Bornean sites (mean Sumatran site rainfall = 3323 mm; Borneo = 3511 mm), which does not support the prediction that higher rainfall should lead to higher plant productivity, although above 2500 mm this effect might be absent [4].

Because the data for these comparisons were compiled post-hoc from a large number of sites where research questions differed, it is important to discuss whether possible differences in research methodology could have influenced the results presented in this paper. Although the data collection between sites differed to the extent that the number of fruits on trees were sometimes scored in different classes, the data always clearly indicate whether fruits were absent or present on a tree and, as such, these data were directly comparable at this level. Another potential bias might be that sampling periods did not always overlap in time and as such might not be directly comparable. Especially since fruit production can be tied to climatic factors such as ENSO [5], [33], systematic differences between Sumatra and Borneo might have confounded comparisons (e.g. due to global climate change). However, for our dataset, the midpoints of the sampling periods did not differ significantly [39]. In addition, a previous analysis between Ketambe and Gunung Palung indicated that for the same time period fruit production is still significantly higher for the Sumatran site [54]. We also attempted to correct for this possible confounding factor by comparing fruit production in three different periods of fruit production. This also reduced biases that may have been introduced through the use of data sets of different durations. Thus, we are confident that our results indeed reflect island differences in fruit production rather than climatically-mediated temporal differences.

It is important to note that our study considered differences in fruiting frequency, and did not incorporate indices of fruit crop size. We suggest that future studies use standardized data collection methods that incorporate crop size measures to permit more detailed future comparisons or conduct analyses at species or community levels (e.g. [55]). Another useful next step in refining comparisons would be to categorise trees as mast- or non mast-type (cf. [56]) and then conduct analyses separately for the two categories. Unfortunately, the duration of data collection at many of the sites included in these analyses did not allow for such an analysis.

These results support a growing body of studies that indicates higher production of forests in Sumatra than on Borneo. These studies show that primate biomass in general, but also specifically for certain species, such as orangutans, is higher on Sumatra than Borneo [39], [40], [43]. Several comparisons also indicate that mammal body size is smaller on Borneo than on Sumatra [48]–[51], which could be the result of an evolution towards smaller body size in an area where resource availability is reduced [32].

Future comparisons of forest production should ideally be conducted by using standardized litterfall protocols, because these yield standard data on actual productivity (kg/ha: e.g. [14], [50], [57]). In combination with collecting litterfall data soil nutrient studies should be conducted that use standard methods to assess soil fertility (e.g. [8]). These studies should ideally also examine productivity measures of similar families, genera or even species on both islands to exclude the influence of island differences in those on the overall analyses. Such future studies will be important in making more fine-grained comparisons between and within the two islands and will help us to better understand the differences in fauna between the islands.

## Materials and Methods

Tree fruit phenology data were collected at two study sites on Sumatra and four sites on Borneo (Figure 1, Table 1). All study sites consisted of undisturbed forest. To ensure that our analyses were not biased by comparing different forest types at different sites, all comparisons were done between forests of the same general type. For this analysis we recognized three broad forest types: peat forest (peat), riverine forest (river), and dryland forest (dry). Peat forests are found on relatively acidic (4<pH<6), nutrient-poor, poorly-drained soils that are overlain by variable amounts of organic matter. Tree species diversity in most Asian peat forests is impoverished relative to more well-drained forest types, and the canopy is relatively low and even [40], [58]. Riverine forest, as defined here, encompasses freshwater swamps and frequently inundated alluvial fans. Soils in riverine forest are generally less acidic than peat soils (pH>6) nutrient rich, seasonally flooded, and poorly drained [59]. Dryland forests, as defined here, are found up to 500 m asl on generally well-drained soils. Species diversity in these dryland forests is relatively high and the canopy is tall and well-structured [58]. Although each of these three forest types encompasses a range of variation due to microhabitat heterogeneity, edaphic effects, and differences in rainfall, this broad classification scheme permitted the comparison of sites that are similar in drainage, species diversity, and structure.

### Study Sites

#### Sumatra

Ketambe (KET) (3°41′N, 97°39′E) is located in the upper Alas valley in Gunung Leuser National Park, Leuser Ecosystem. This study area mainly consists of primary dryland rain forest and was described in detail by Rijksen [60], van Schaik and Mirmanto [9] and Wich and van Schaik [5].

Suaq Balimbing (SB) (3°04′N, 97°26′E) is located in the western coastal plain, and consists of a variety of floodplain and hill forest habitats. It forms part of Gunung Leuser National Park, Leuser Ecosystem [5].

#### Borneo

Barito Ulu (BU) is located in Central Kalimantan, Indonesia, at 114°0′E, 0°06′S. The research area covers 430 ha and contains a mosaic of forest types. These include several types of tropical lowland evergreen rain forest [35], [61].

Gunung Palung (GP) (1°13′S, 110°7′E) is located in Gunung Palung National Park, West Kalimantan, Indonesia. Data were collected in several distinct forest types at the Cabang Panti Research Station: peat swamp (5–10 m a.s.l.), riverine forest (freshwater swamp; 5–10 m a.s.l.), and three types of dryland forest (alluvial bench, lowland sandstone, and lowland granite; 5–400 m a.s.l.). General descriptions and detailed data on the plant composition of each habitat are provided in Webb [62], Cannon and Leighton [63], Marshall [59], and Paoli *et al.* [64], [65].

Sungai Wain (SW) is located in Sungai Wain Protected Forest, East Kalimantan, Indonesia (1°05′S, 116°49′E) and consists of lowland dipterocarp forest. The topography of the reserve consists of gentle to sometimes steep hills, and is intersected by many small rivers [66].

Tanjung Puting (TP) (2°46′S, 111°52′E) is located in Tanjung Puting National Park, Central Kalimantan, Indonesia. Data were collected at Natai Lengkuas Station in peat swamps that were periodically flooded with freshwater [67].

#### Field Methods

Trained observers collected monthly presence/absence of fruit (immature and/or mature, cf. [56]) on each tree by using binoculars to examine the canopy of each tree.

#### Analyses

For each site we calculated the percentage of trees fruiting per month and included all trees with a diameter at breast height (DBH) larger than 10 cm (Table S1, S3, S7a–c, Data S1). At all size most trees in the dataset were identified to the species level or morphospecies level. Since the datasets varied in duration (Table 1) we divided fruit production into three classes for comparison using the following procedure. All monthly percentage scores were standardised by calculating standardised deviates per site/forest type combination (or z-scores: [68], which is the monthly value minus the mean divided by the standard deviation. Months with a z-score<−1 were classified as low fruit periods (LFP), a z-score between −1 and 1 and medium fruit periods (MFP), and z-scores>1 as high fruit periods (HFP).

Because observations over months within a site are correlated over time and not independent, we fit time-series models for each site (Methods S1). These time series patterns influence the variability of estimators such as the mean availability of fruit per month for each site. All models included effects for fruiting level (LFP, MFP, HFP) and DBH (diameter at breast height (1.3 m)) category (defined in Table 1). The time series error structure for each site was selected using the smallest AIC criterion value for the fit to the site (Methods S1). Potential error structures included independence, autoregressive (AR) of order up to 4, moving average (MA) of order up to 4, and ARMA of orders up to (2,2) [69]. Each model provides estimates of fruit production for each fruiting level and DBH category observed at a given site. Time series models were fit to measurements on the original scale.

For forest type comparisons between sites in Borneo and Sumatra, estimates from the time series models within a fruiting level by DBH category were directly averaged within habitat. In order to provide a single summary for Sumatra versus Borneo, an average for each site was produced by averaging its estimates by fruit and DBH categories. Averages were computed according to the distribution of measurements across fruiting level by DBH category within each site. The resulting standard error for the average within a site takes into account the covariance among estimates within a site. The estimates of the sites within each island were then averaged. Satterthwaite approximations were used to determine degrees of freedom, which is a more conservative procedure than directly pooling degrees of freedom. The Satterhwaite approximation for degrees of freedom is appropriate when taking linear combinations of sample variances [70], [71]. Neter et al. [72] and Rice [73] give the formula as df = (MS1+MS2)∧2/(MS1∧2/df1+MS2∧2/df2), where MS1 and MS2 are two estimates of variance and df1 and df2 are the degrees of freedom associated with the variance estimates. This formula does not assume that the two variances are equal as would a pooled variance estimate formula. Using these average estimates, we conducted pairwise comparisons between sites on different islands in similar forest types. For these analyses, we corrected the significance level by multiplying the p-value by 13, the number of comparisons. Results when models were selected using the Bayesian information criterion (BIC) were analogous to the presented results.

Means and standard error are reported in the text for statistical analyses unless otherwise indicated. We ran all analyses in R version 2.12.1 [74]. All probability levels are two-tailed, and the significance for all tests was set at alpha <0.05 unless noted. The R package used was nlme (Linear and Nonlinear Mixed Effects Models) with the commands gls (generalized least squares) and corARMA (ARMA(p,q) Correlation Structure).

## Supporting Information

Methods S1 Details on the methods and models used.
(DOC)

Data S1 Raw data used for analyses.
(XLS)

Table S1 Counts of observations and time series estimated mean differences between Sumatra and Borneo riverine forest habitats.
(DOC)

Table S2 Differences in time series estimated fruit production for riverine forests.
(DOC)

Table S3 Counts of observations in peat swamp forests.
(DOC)

Table S4 Differences in time series estimated fruit production in peat forests.
(DOC)

Table S5 Comparison of time series estimated differences in fruit production (% fruiting) at Suaq Balimbing in Sumatra and Gunung Palung in Borneo peat swamp habitats (model includes time series correction, fruit level, and DBH).
(DOC)

Table S6 Comparison of time series estimated differences in fruit production (% fruiting) at Suaq Balimbing in Sumatra and Tanjung Puting in Borneo for peat swamp habitats (model includes time series correction, fruit level, and DBH).
(DOC)

Table S7 Counts of observations during low, middle, and high fruit periods in Sumatra and Borneo dryland forest sites.
(DOC)

Table S8 Differences in time series estimated fruit production in dryland forests.
(DOC)
