# Supplementary material for: Forest Fruit Production Is Higher on Sumatra Than on Borneo
Source: PLoS One. 2011 Jun 28;6(6):e21278. doi: 10.1371/journal.pone.0021278 (PMC3125178; doi:10.1371/journal.pone.0021278)
Supplement: Methods S1 — Details on the methods and models used. (DOC) [file pone.0021278.s001.doc]

**Supporting information**

***Supplemental Methods***

**RIVERINE FORESTS**

In Sumatra, data were gathered from 1994-1999 at Suaq Balimbing. In Borneo, data were gathered at Gunung Palung from 1985-1991. Observations at these sites are classified by the diameter of the tree (15-29.9 to 90+) and fruit level (low, medium, high).

Time series models for riverine habitats were fit separately to Suaq Balimbing in Sumatra and Gunung Palung in Borneo sites. Models included a factor for tree diameter, fruit level, and a tree diameter-fruit level interaction. Time was included through an autoregressive (AR) model of order up to 4, a moving average (MA) model of order up to 4, or an ARMA model of orders up to (2,2) on the residuals (Cowpertwait and Metcalfe 2009). In all cases, models with the tree diameter-fruit level interaction fit better (according to the AIC criterion) than models with only tree diameter and fruit level main effects.   For Gunung Palung, the linear model with a MA(2) time series structure had the best AIC fit criterion value.  For Suaq Balimbing, a time series structure with a lag 1 autocorrelation (an AR(1) model) had the best AIC fit criterion value. For Suaq Balimbing, a time series structure with a lag 1 autocorrelation (an AR(1) model) was preferred. From both model fits, estimates of the average fruit abundance within fruit level and tree diameter class along with standard errors of the estimates were produced (Figure 3). Counts of observations in riverine forests are presented in Table S1 whereas the other fruit production results are presented in Table S2.

Table S1

Table S2

**PEAT SWAMPS**

In Sumatra, data were gathered from 1994-1999 at Suaq (66 months). In Borneo, data were gathered at Gunung Palung from 1985-1991 (79 months) and at Tanjung Putung from 1993-1996 (40 months). Observations at these sites are classified by the diameter of the tree (15-29.9 to 90+) and fruit level (low, medium, high; Table S3).

Models were fit to the three peat swamp habitat forests: Suaq Balimbing in Sumatra and Gunung Palung and Tanjung Putting in Borneo and GP PS. The AIC best fit models for Suaq and Tanjung Putting were ARMA (2,2) models. The AIC best fit model for Gunung Palung was an AR(3) model. Estimated differences, standard errors, t-values, and p-values for comparing Suaq in Sumatra to Gunung Palung in Borneo are provided below (Table S4). All differences favour Sumatra and most are statistically significant. Estimated differences, standard errors, t-values, and p-values for comparing Suaq Balimbing in Sumatra to Tanjung Puting in Borneo are provided below (Table S5 and S6).

Table S3

Table S4

Table S5

Table S6

**DRYLAND FOREST**

Data came from seven sites classified as dryland forest: two in Sumatra and five in Borneo. In Sumatra, data were gathered at Ketambe (1988-2001) and Suaq (1994-1999). In Borneo, data were gathered at three sites within Gunung Palung (1985 – 1991), Barito Ulu (1992-2000), and Sungai Wain (1998-2002). The three tables below give counts of low, mid, and high fruit observations for combinations of tree diameter and site for seven sites (Tables 7a-c). It is noteworthy that some sites only had observations on small trees, whereas others had observations on trees across the size range. Ketambe and Suaq in Sumatra and the three sites in Gunung Palung in Borneo had observations across the full range of tree sizes. Barito Ulu in Borneo had observations only on small trees. Sungai Wain in Borneo had observations on small and moderate sized trees. The size of trees has been correlated with the number of fruit produced on a tree [1]. Thus, we compare island fruit production (Sumatra vs. Borneo) within tree diameter class at sites with a given tree profile (full range of trees, smaller trees only) in addition to making a comparison using all the sites. In addition, we include the results of analyses by fruit level and DBH size category.

Table S7a-c

Table S8

1. Chapman CA, Chapman LJ, Wrangham R, Hunt K, Gebo D, Gardner L (1992) Estimators of fruit abundance of tropical trees. Biotropica 24: 527-531.
