# Supplementary material for: Forest Fruit Production Is Higher on Sumatra Than on Borneo
Source: PLoS One. 2011 Jun 28;6(6):e21278. doi: 10.1371/journal.pone.0021278 (PMC3125178; doi:10.1371/journal.pone.0021278)
Supplement: Table S1 — Counts of observations and time series estimated mean differences between Sumatra and Borneo riverine forest habitats. (DOC) [file pone.0021278.s003.doc]

Table S1. Counts of observations and time series estimated mean differences between Sumatra and Borneo riverine forest habitats.

|  |  | Counts of observations | | | | | | Estimated mean % fruiting difference | | |
| --- | --- | --- | --- | --- | --- | --- | --- | --- | --- | --- |
|  | Site (Island) | Suaq (Sumatra) | | | Gunung Palung (Borneo) | | | Sumatra minus Borneo | | |
|  | Fruit level | Low | Mid | High | Low | Mid | High | Low | Mid | High |
| Tree Diameter | 15-29.9 | 14 | 10 | 10 | 10 | 60 | 9 | 2.23 | 4.33 | 5.78 |
|  | 30-44.9 | 12 | 9 | 9 | 9 | 59 | 11 | 2.63 | 5.81 | 9.89 |
|  | 45-59.9 | 4 | 10 | 10 | 10 | 58 | 11 | 3.82 | 7.43 | 9.91 |
|  | 60-74.9 |  |  |  |  | 63 | 16 |  |  |  |
|  | 76-89.9 |  |  |  |  | 65 | 11 |  |  |  |
