# Supplementary material for: Forest Fruit Production Is Higher on Sumatra Than on Borneo
Source: PLoS One. 2011 Jun 28;6(6):e21278. doi: 10.1371/journal.pone.0021278 (PMC3125178; doi:10.1371/journal.pone.0021278)
Supplement: Table S2 — Differences in time series estimated fruit production for riverine forests. (DOC) [file pone.0021278.s004.doc]

Table S2. Differences in time series estimated fruit production for riverine forests.

| Fruit level | Diameter | Estimated difference in % fruiting  (Sumatra-Borneo) | Standard error | t statistic | P-value (two-sided) |
| --- | --- | --- | --- | --- | --- |
| Low | 15-29.9 | 2.23 | 1.63 | 1.37 | 0.1711 |
| Low | 30-44.9 | 2.63 | 1.66 | 1.58 | 0.1148 |
| Low | 45-59.9 | 3.82 | 2.23 | 1.71 | 0.0888 |
| Mid | 15-29.9 | 4.33 | 1.03 | 4.19 | <0.0001 |
| Mid | 30-44.9 | 5.81 | 1.04 | 5.61 | <0.0001 |
| Mid | 45-59.9 | 7.43 | 1.00 | 7.41 | <0.0001 |
| High | 15-29.9 | 5.78 | 2.01 | 2.88 | 0.0043 |
| High | 30-44.9 | 9.89 | 1.89 | 5.24 | <0.0001 |
| High | 45-59.9 | 9.91 | 1.88 | 5.28 | <0.0001 |

Note: The riverine forest sites are Suaq in Sumatra and Gunung Palung in Borneo.
