# Supplementary material for: Forest Fruit Production Is Higher on Sumatra Than on Borneo
Source: PLoS One. 2011 Jun 28;6(6):e21278. doi: 10.1371/journal.pone.0021278 (PMC3125178; doi:10.1371/journal.pone.0021278)
Supplement: Table S3 — Counts of observations in peat swamp forests. (DOC) [file pone.0021278.s005.doc]

Table S3. Counts of observations in peat swamp forests.

| Count of observations | Island | Sumatra | | | Borneo | | | | | |
| --- | --- | --- | --- | --- | --- | --- | --- | --- | --- | --- |
|  | Site | SBps | | | GP PS | | | TNPUT | | |
|  | Fruit level | Low | Mid | High | Low | Mid | High | Low | Mid | High |
| Tree Diameter | 15-29.9 | 11 | 45 | 9 | 11 | 57 | 11 | 8 | 26 | 6 |
|  | 30-44.9 | 5 | 48 | 12 | 10 | 56 | 13 | 7 | 27 | 6 |
|  | 45-59.9 | 6 | 47 | 12 | 10 | 60 | 9 | 7 | 31 | 2 |
|  | 60-74.9 | 12 | 37 | 16 | 19 | 55 | 5 |  |  |  |
|  | 76-89.9 |  |  |  |  | 61 | 18 |  |  |  |
