# Supplementary material for: Forest Fruit Production Is Higher on Sumatra Than on Borneo
Source: PLoS One. 2011 Jun 28;6(6):e21278. doi: 10.1371/journal.pone.0021278 (PMC3125178; doi:10.1371/journal.pone.0021278)
Supplement: Table S4 — Differences in time series estimated fruit production in peat forests. (DOC) [file pone.0021278.s006.doc]

Table S4. Differences in time series estimated fruit production in peat forests.

| Fruit level | Diameter | Mean difference in % fruiting  (Sumatra-Borneo) | Standard error | t statistic | P value (two-sided) |
| --- | --- | --- | --- | --- | --- |
| Low | 15-29.9 | 0.44 | 1.72 | 0.25 | 0.7991 |
| Low | 30-44.9 | 9.86 | 2.42 | 4.08 | 0.0001 |
| Low | 45-59.9 | 18.29 | 2.00 | 9.15 | p < 0.0001 |
| Low | 60-74.9 | 45.45 | 1.95 | 23.34 | p < 0.0001 |
| Mid | 15-29.9 | 2.42 | 1.14 | 2.12 | 0.0349 |
| Mid | 30-44.9 | 16.41 | 1.12 | 14.65 | p < 0.0001 |
| Mid | 45-59.9 | 22.69 | 1.12 | 20.33 | p < 0.0001 |
| Mid | 60-74.9 | 48.86 | 1.36 | 35.80 | p < 0.0001 |
| High | 15-29.9 | 2.74 | 1.90 | 1.44 | 0.1499 |
| High | 30-44.9 | 22.63 | 1.75 | 12.95 | p < 0.0001 |
| High | 45-59.9 | 23.78 | 1.94 | 12.24 | p < 0.0001 |
| High | 60-74.9 | 39.89 | 2.36 | 16.91 | p < 0.0001 |

Note: The peat swamp sites are Suaq in Sumatra and Gunung Palung and Tanjung Puting in Borneo.
