# Supplementary material for: Forest Fruit Production Is Higher on Sumatra Than on Borneo
Source: PLoS One. 2011 Jun 28;6(6):e21278. doi: 10.1371/journal.pone.0021278 (PMC3125178; doi:10.1371/journal.pone.0021278)
Supplement: Table S5 — Comparison of time series estimated differences in fruit production (% fruiting) at Suaq Balimbing in Sumatra and Gunung Palung in Borneo peat swamp habitats (model includes time series correction, fruit level, and DBH). (DOC) [file pone.0021278.s007.doc]

Table S5. Comparison of time series estimated differences in fruit production (% fruiting) at Suaq Balimbing in Sumatra and Gunung Palung in Borneo peat swamp habitats (model includes time series correction, fruit level, and DBH).

| Fruit level | Diameter | Estimated difference | Standard error | T statistic | P value (2 sided) |
| --- | --- | --- | --- | --- | --- |
| Low | 15-29.9 | 2.28 | 1.99 | 1.15 | 0.25 |
| Low | 30-44.9 | 9.87 | 2.65 | 3.72 | p<0.0001 |
| Low | 45-59.9 | 17.18 | 2.26 | 7.60 | p<0.0001 |
| Low | 60-74.9 | 45.45 | 1.95 | 23.34 | p<0.0001 |
| Mid | 15-29.9 | 4.65 | 1.30 | 3.57 | p< 0.001 |
| Mid | 30-44.9 | 16.02 | 1.29 | 12.46 | p<0.0001 |
| Mid | 45-59.9 | 23.05 | 1.28 | 18.02 | p<0.0001 |
| Mid | 60-74.9 | 48.86 | 1.36 | 35.80 | p<0.0001 |
| High | 15-29.9 | 4.50 | 2.17 | 2.07 | 0.04 |
| High | 30-44.9 | 21.05 | 2.00 | 10.54 | p<0.0001 |
| High | 45-59.9 | 24.53 | 2.20 | 11.12 | p<0.0001 |
| High | 60-74.9 | 39.89 | 2.36 | 16.91 | p<0.0001 |
