# Supplementary material for: Forest Fruit Production Is Higher on Sumatra Than on Borneo
Source: PLoS One. 2011 Jun 28;6(6):e21278. doi: 10.1371/journal.pone.0021278 (PMC3125178; doi:10.1371/journal.pone.0021278)
Supplement: Table S6 — Comparison of time series estimated differences in fruit production (% fruiting) at Suaq Balimbing in Sumatra and Tanjung Puting in Borneo for peat swamp habitats (model includes time series correction, fruit level, and DBH). (DOC) [file pone.0021278.s008.doc]

Table S6. Comparison of time series estimated differences in fruit production (% fruiting) at Suaq Balimbing in Sumatra and Tanjung Puting in Borneo for peat swamp habitats (model includes time series correction, fruit level, and DBH).

| Fruit level | Diameter | Estimated difference | Standard error | T statistic | P value (2 sided) |
| --- | --- | --- | --- | --- | --- |
| Low | 15-29.9 | -1.41 | 1.69 | -0.83 | 0.41 |
| Low | 30-44.9 | 9.85 | 2.40 | 4.11 | p<0.0001 |
| Low | 45-59.9 | 19.39 | 1.99 | 9.80 | p<0.0001 |
| Mid | 15-29.9 | 0.19 | 1.12 | 0.17 | 0.87 |
| Mid | 30-44.9 | 16.80 | 1.09 | 15.35 | p<0.0001 |
| Mid | 45-59.9 | 22.33 | 1.09 | 20.53 | p<0.0001 |
| High | 15-29.9 | 0.97 | 1.89 | 0.51 | 0.61 |
| High | 30-44.9 | 24.22 | 1.76 | 13.76 | p<0.0001 |
| High | 45-59.9 | 23.04 | 2.10 | 10.98 | p<0.0001 |
