# Supplementary material for: Forest Fruit Production Is Higher on Sumatra Than on Borneo
Source: PLoS One. 2011 Jun 28;6(6):e21278. doi: 10.1371/journal.pone.0021278 (PMC3125178; doi:10.1371/journal.pone.0021278)
Supplement: Table S7 — Counts of observations during low, middle, and high fruit periods in Sumatra and Borneo dryland forest sites. (DOC) [file pone.0021278.s009.doc]

Table S7a-c. Counts of observations during low, middle, and high fruit periods in Sumatra and Borneo dryland forest sites.

a)

| *Low fruit* observations | Island | Sumatra | | Borneo | | | | |
| --- | --- | --- | --- | --- | --- | --- | --- | --- |
|  | Site | Ketambe | Sb hill | GP AB | GP LG | GP LS | BU | SW |
| Tree Diameter | 15-29.9 | 25 | 13 | 9 | 15 | 13 | 17 | 8 |
|  | 30-44.9 | 20 | 16 | 10 | 12 | 14 |  |  |
|  | 45-59.9 | 24 | 16 | 19 | 14 | 12 |  |  |
|  | 60-74.9 | 21 | 21 | 12 |  | 16 |  |  |
|  | 76-89.9 | 14 |  |  |  | 26 |  |  |
|  | 90+ | 19 |  |  |  | 21 |  |  |

b)

| *Mid fruit* observations | Island | Sumatra | | Borneo | | | | |
| --- | --- | --- | --- | --- | --- | --- | --- | --- |
|  | Site | Ketambe | Sb hill | GP AB | GP LG | GP LS | BU | SW |
| Tree Diameter | 15-29.9 | 101 | 44 | 56 | 51 | 54 | 86 | 39 |
|  | 30-44.9 | 107 | 41 | 57 | 58 | 50 | 94 | 42 |
|  | 45-59.9 | 100 | 36 | 47 | 56 | 53 |  | 42 |
|  | 60-74.9 | 118 | 40 | 57 | 67 | 50 |  | 44 |
|  | 76-89.9 | 136 | 0 | 63 | 65 | 40 |  |  |
|  | 90+ | 119 | 52 | 65 | 58 | 44 |  |  |

c)

| *High fruit* observations | Island | Sumatra | | Borneo | | | | |
| --- | --- | --- | --- | --- | --- | --- | --- | --- |
|  | Site | Ketambe | Sb hill | GP AB | GP LG | GP LS | BU | SW |
| Tree Diameter | 15-29.9 | 26 | 9 | 14 | 13 | 12 | 18 | 6 |
|  | 30-44.9 | 25 | 9 | 12 | 9 | 15 | 27 | 11 |
|  | 45-59.9 | 28 | 14 | 13 | 9 | 14 |  | 11 |
|  | 60-74.9 | 13 | 5 | 10 | 12 | 13 |  | 9 |
|  | 76-89.9 | 2 | 0 | 16 | 14 | 13 |  |  |
|  | 90+ | 14 | 14 | 14 | 18 | 11 |  |  |
