# Supplementary material for: Forest Fruit Production Is Higher on Sumatra Than on Borneo
Source: PLoS One. 2011 Jun 28;6(6):e21278. doi: 10.1371/journal.pone.0021278 (PMC3125178; doi:10.1371/journal.pone.0021278)
Supplement: Table S8 — Differences in time series estimated fruit production in dryland forests. (DOC) [file pone.0021278.s010.doc]

Table S8. Differences in time series estimated fruit production in dryland forests.

| Fruit level | Diameter | Mean difference in % fruiting  (Sumatra-Borneo) | Standard error | t statistic | DF | p-value (2 sided) |
| --- | --- | --- | --- | --- | --- | --- |
| Low | 15-29.9 | 0.74 | 1.59 | 0.47 | 1128 | 0.64 |
| Low | 30-44.9 | 3.36 | 1.79 | 1.88 | 1303 | 0.06 |
| Low | 45-59.9 | 5.72 | 1.68 | 3.41 | 1203 | p < 0.0001 |
| Low | 60-74.9 | 0.86 | 1.66 | 0.52 | 997 | 0.60 |
| Low | 75-89.9 | 15.12 | 2.32 | 6.53 | 1077 | p < 0.0001 |
| Low | 90 | 13.38 | 2.14 | 6.26 | 1147 | p < 0.0001 |
| Mid | 15-29.9 | 2.41 | 0.95 | 2.54 | 1047 | 0.01 |
| Mid | 30-44.9 | 9.26 | 0.94 | 9.85 | 1036 | p < 0.0001 |
| Mid | 45-59.9 | 12.12 | 1.00 | 12.15 | 1000 | p < 0.0001 |
| Mid | 60-74.9 | 12.55 | 0.97 | 13.00 | 979 | p < 0.0001 |
| Mid | 75-89.9 | 39.19 | 1.26 | 31.10 | 1732 | p < 0.0001 |
| Mid | 90 | 14.80 | 1.03 | 14.31 | 1038 | p < 0.0001 |
| High | 15-29.9 | 6.81 | 1.90 | 3.59 | 1088 | p < 0.0001 |
| High | 30-44.9 | 13.16 | 1.90 | 6.94 | 1065 | p < 0.0001 |
| High | 45-59.9 | 11.07 | 1.90 | 5.83 | 1368 | p < 0.0001 |
| High | 60-74.9 | 14.21 | 2.20 | 6.47 | 1042 | p < 0.0001 |
| High | 75-89.9 | 40.47 | 4.90 | 8.26 | 1117 | p < 0.0001 |
| High | 90 | 23.75 | 2.01 | 11.83 | 1153 | p < 0.0001 |

Note: Analyses included Ketambe and Suaq Balimbing in Sumatra and Gunung Palung (3 sites), Barito Ulu, and Sungai Wain in Borneo.
